# Self-Assessed Digital Competences of Romanian Teachers During the COVID-19 Pandemic

**DOI:** 10.3389/fpsyg.2022.810359

**Published:** 2022-03-01

**Authors:** Adrian Hatos, Mirela-Lăcrimioara Cosma, Otilia Clipa

**Affiliations:** ^1^Department of Sociology, University of Oradea, Oradea, Romania; ^2^Department of Science of Education, Stefan cel Mare University, Suceava, Romania

**Keywords:** digital competences, teachers perceived digital skills, Romanian education, teachers, teachers training

## Abstract

Studies on the determinants of school results have shown that they depend largely on the context of learning. Concerning the pandemic, teachers have been forced to find online teaching methods, which leads us to the central issue of this study of whether the effectiveness of online education depends on teachers’ digital skills. Therefore, in this study, we analyzed the perceived digital competences of Romanian pre-tertiary cycle teachers about their professional status, school location, gender, age, taught field, and prior participation in training for online teaching. Using data from 3,419 self-completed questionnaires in an online survey of teachers performed at the beginning of the global lockdown in March 2020, we have built two reliable measures of perceived digital skills, namely the Self-Assessed Multimedia and Online Skills Score (SMOS) and the Self-Assessed Digital Office Skills Score (SDOS), which were the dependent variables in our study. Hierarchical linear regressions were used to test the hypotheses regarding the variations of dependent variables, measuring the two concepts of self-assessed digital skills (SMOS and SDOS). These concepts underlined that both decrease with age and are positively affected by prior attendance at training sessions for online teaching skills and by having ICT and informatics as a taught subject field. However, teachers of all specialties are relatively significantly less skilled in this field. The most important results concern the impact of gender and professional status on the teachers’ self-assessed digital competences. In summary, it appears that self-assessed office digital skills are a specialism demonstrated mainly by female teachers, while multimedia and online skills are perceived by teachers to be a “male” domain. Simultaneously, net of the other variables, a higher status within the teaching profession correlates positively with perceived office digital skills. Lastly, implications for future research, as well as for educational interventions and policies, are discussed.

## Introduction

Fundamentally, all education systems aim to shape Students’ personalities, knowledge, and skills into their very best forms. During the pandemic period, these core aims were significantly challenged by schools’ closures and the generalized shift to digital means of communication and information-sharing (Ali and Kaur, 2020; Schleicher, 2020). For the first time in decades of technological advances, entire societies were forced to maximize their ICT infrastructure and abilities to meet the basic objectives of schooling, and other societal needs OECD, 2020a, b; Maněnová et al., 2021).

The impact of the use of technology on student outcomes is dependent on its integration in the classroom to support teaching and learning practices. Teachers’ digital competences are crucial to optimize new technologies in the classroom (Engen and Engen, 2019), therefore, it is important to understand that teachers’ digital skills are related to Students’ performance in digital learning conditions (OECD, 2019a), in this case, the COVID-19 pandemic.

Highly digitally skilled teachers cannot only do a better job in creating and distributing content as well as in communicating with their pupils, but they can also increase the levels of academic achievement. This rich communication from teacher to student is critical for student outcomes: studies have shown that students who have high levels of engagement have better grades and display better personal conduct, as well as higher levels of self-esteem and compliance with socially appropriate behaviors (Lam et al., 2014). Furthermore, it is associated with high rates of school completion (Veiga et al., 2012).

Perceived digital skills, of teachers and students alike, have played a crucial role during this pandemic period as an individual’s assessment of their ICT skills is a significant mediator in terms of how effectively they are put into practice (Winstone et al., 2021).

However, for entire systems of education, including the Romanian system, it was a huge challenge to rapidly shift from on-site teaching and learning to online didactic activities (Lim, 2020), using technology to facilitate and enhance Students’ performance and wellbeing. Issues of access (e.g., access to computers, internet, and even digital skills development courses), along with gaps in both Students’ and teachers’ digital skills and a lack of confidence or overconfidence inabilities of both students and teachers raised formidable obstacles against the delivery of education during the pandemic (Ali and Kaur, 2020; Schleicher, 2020). On the other hand, this uniquely challenging period allowed teachers to develop their digital skills and practice teaching by utilizing apps, software, videos, and films, when the online environment was the only option for keeping in touch with their students (Saavedra, 2020).

In this article, we focus on one of the aspects most critical for teachers performing in online education: their perceived digital skills. To meet this objective, we build several hypotheses concerning teachers’ self-perceived digital skills and test them on a large data set produced through an online survey carried out during the pandemic.

The paper will discuss the context of Romanian education and access to ICT in Romanian schools, then the concept of teachers’ perceived digital skills (including their relationships with actual digital performativity and how it is operationalized for the specific case of online education). Subsequently, a set of hypotheses about the variation of teachers’ digital skills will be identified, based on a review of the existing literature. Then, in the empirical part of the article, we will discuss our two indices of perceived digital skills and the results of hierarchical multiple regressions in which these two indices are used as dependent variables to test our hypotheses.

### The Relevance of Teachers Perceived and Actual Digital Skills for Student Achievement *via* Online Education

Students’ academic results are dependent on contextual factors, such as educational materials, the educational and cultural background of a Student’s family, how students spend their free time, and other psycho-climatic factors. It is almost self-evident that the effectiveness of online education is significantly dependent on teachers’ digital competences. Innovative teaching using ICT requires much more than basic ICT skills. Teachers have the power to transform ICT in learning and communication technology, as teachers’ perceived usefulness, information processing skills (the skills of information access, information usage, and information management), and information ethics could predict teachers’ competence to develop Students’ information literacy (Wu et al., 2022); however, both teachers and their students must first realize the potential of ICT to impact learning and to transform education (Napal-Fraile et al., 2018).

Researchers highlighted the importance of teachers’ digital competences, computer provisions, and electronic devices in online education, arguing that ICT is facilitating the establishment of a skilled community and workforce for a knowledge society (Malik, 2018). As such, we can assume that the higher the level of teachers’ digital skills, the easier the information transfer. Likewise, the higher the volume of information acquired, the more accurate the appreciation.

More recently, Røkenes and Krumsvik (2014) reviewed a vast number of studies on teachers’ use of ICT in the classroom, revealing that the effectiveness of implementing ICT in schools may partly rely on Students’ digital competence (Røkenes and Krumsvik, 2014; Wu et al., 2022) as well as on how effectively teachers can implement and use ICT for teaching and learning. Indeed, strong correlations have been found between teachers’ digital competence and Students’ subject learning outcomes in Norwegian secondary schools (Røkenes and Krumsvik, 2014). ICT in education can be used for a variety of different purposes, such as active teaching and learning through Students’ involvement (Ghavifekr and Quan, 2020), improving Students’ understanding of key concepts or developing content knowledge and specific abilities, as well as a correlating improvement in their learning results (Furman et al., 2019). In this regard, Isteniè (2021) builds a strong argument that feedback with a supportive function is essential in a time when students and teachers are working remotely.

### The Importance of Attitudes Toward Information and Communication Technologies and Perceived Information and Communication Technologies Skills

Regarding technology adoption in the classroom, some studies have shown that the successful implementation of educational technologies is dependent on the attitudes of educators toward ICT, who eventually determine how they are used in their teaching practice (Asad et al., 2020; Ifinedo et al., 2020; Hofer et al., 2021). In this context, teachers’ perceived ICT competences play a significant role. Birgin et al. (2020) highlighted that educators’ attitudes toward computer technologies are also related to their perceived computer competence. Furthermore, teachers perceive digital competence as a significant predictor of their attitudes toward computers (Lucas et al., 2021). In the same manner, researchers illustrated how several educators, whose perceived computer competence was low, also showed negative or neutral attitudes toward the use of ICT in education in general. Moreover, it has been found that the more highly teachers rate their digital competence, the more likely they are to use ICT in their work (Sundqvist et al., 2020), as illustrated by Malaysian teachers whose digital competency and confidence level in using ICT are in a positive relationship (Tasir et al., 2012). However, in other studies, the limitations in teachers’ ICT knowledge have caused anxiety about using ICT in the classroom, and thus, they are not confident in using it to teach (Arkorful et al., 2021; Huang et al., 2021; Šabić et al., 2021), namely in front of a class of children who are perhaps more digitally literate than they are (Van Mechelen et al., 2021). Keeping this in mind, teachers, who are not confident in using ICT in their teaching, will encounter difficulties in preparing their students to be confident in the use of ICT for themselves (Starčič et al., 2016), but at the same time, as Willems et al. (2021) found out, pre−service teachers’ self-regulation and mastery approach goals are strengthened when using case studies (even examples) that are authentic.

### Context: The Advance of Digitalization and Internet Penetration Rate in Romania

**FIGURE 1:**
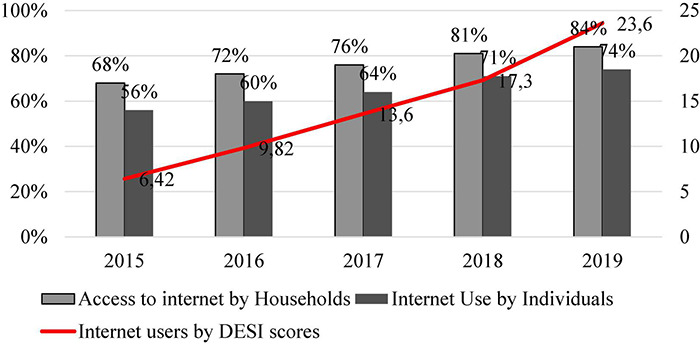
Internet penetration in Romania in the last 5 years (DESI, 2018a; Eurostat, 2020). The Digital Economy and Society Index (DESI) is a composite index that summarizes relevant indicators on Europe’s digital performance and tracks the evolution of EU Member States in digital competitiveness. DESI Indicators: connectivity, human capital, use of internet services, integration of digital technology, digital public services (DESI, 2018b).

Romania’s internet penetration rate has been increasing, both in terms of access and terms of use, reaching a rate of 80% internet penetration by January 2020 (Hootsuite and Social, 2019). Additionally, the 2018 Digital Economy and Society Index (DESI) score for Romanian internet users shows growth in penetration rates in the last 5 years DESI, 2018a, b). Scores are shown from a minimum of 0 to a maximum of 100, and on this chart, the scores are represented by a line. As illustrated in Figure 1 below, having access to the internet does not necessarily equate to using the internet.

### Digitalization and Internet Penetration in Romanian Schools

Romanian schools have gradually undergone the digitalization process. In 2001, a government program called Computerized Educational System [Sistemul Educaţional Informatizat (SEI)] was launched to computerize the Romanian education system. Another government-level target was to achieve the objectives of the National Plan for European Union membership by equipping all pre-university education institutions with computer laboratories, based on Government Decision No. 1108/25.09.2003 (Noveanu et al., 2008).

The inter-county differences in Romania’s school digitalization processes are closely related to regional development and GDP per capita between the counties. To address these discrepancies, the RO-NET project (MCSI, 2011) was launched in 2011 to build national broadband infrastructure in socio-economically disadvantaged areas by using structural funds. Eurostat (2020) from different regions show a negative correlation between GDP per capita/counties and digitalization process, except for in the West of the country (where GDP per capita is €10,800) and in the Northwest (where GDP per capita is €9,800), as together they sum up several 128 localities who benefit the most from broadband internet. In the poorest region of Romania (the North East according to Eurostat 2018, where GDP per capita is €6,600) 115 localities benefit from broadband internet. In the richest region, Bucharest-Ilfov, according to Eurostat 2018 (where GDP per capita is €24,000), a smaller number of localities benefit from broadband internet (i.e., 84). In 2005, according to the Bologna Process, it was compulsory to include a special course for ICT in education in pre-service teacher training. With increasing access to computers and the internet, teachers began to take advantage of technology to encourage students to learn; however, computer science, as a subject, became mandatory only in 2017, based on an order issued by the Minister of National Education—Ministerul Educaţiei Naţionale (MEN) no. 3393/02.28.2017. In 2019, MEN launched a national project for teachers CRED (55.000 teachers’ participants) who follow the in-service teacher training courses for updating didactical strategy, intending to adopt the national curricula for primary and secondary level and to create the Open Educational Resources. In 2020, the project transformed, with all activities shifted from being on-site to being online and teachers trained to use virtual tools for education, as well as to share these experiences with their colleagues (MEC, 2014-2020).

In summary, in Romania, several initiatives are in place for in-service teacher training in the special and high current areas of the introduction of digital tools in education and increasing the digital competences of Romanian teachers.

## Literature Review

### Concept: Teachers’ Actual Digital Abilities and Perceived Digital Abilities

Bringing digital technology into the classroom means more challenges and responsibilities for teachers. Besides the professional competences that already exist, digital competences have become a new necessity to handle teaching, guidance, and assessment. In this context of teachers’ digital competence, four key concepts highlight the need to handle technology: computer literacy, media literacy, digital literacy, and digital competence (Røkenes and Krumsvik, 2014).

As a general definition, a teacher’s digital competence is their proficiency in using ICT in a professional context with good pedagogical judgment. The focus here is on pedagogy and the subject itself, with technical skills being part of the general digital competence concept (Cabero-Almenara et al., 2020). In the context of digital competence, Janssen et al. (2013) recommend separating use and skills as two facets of digital competence: general vs. “pure” digital competence (i.e., it is difficult to identify what is “pure” digital competence and what is derived from other domains/cognitive processes); digital competence vs. values and attitudes (i.e., ethics and social values are not necessarily considered as a part of digital competence) (Mâţă et al., 2020); and digital competence vs. digital preference. Indeed, experts recommend drawing a clear line between digital competence and personal preference, which is the individual’s choice or desire to use a particular digital technology (Vasileva-Stojanovska et al., 2015).

More specifically, digital abilities are classified by Van Dijk (2020) as operational abilities (i.e., operating hardware, software, and networks); formal abilities (i.e., understanding and managing formal characteristics of a computer and a network, as well as their structures: files and hyperlinks); informational abilities (i.e., searching, selecting processing, and evaluating information from specific sources on computers and networks); and strategic abilities (i.e., using the afore-mentioned information as a way to achieve specific objectives for improving someone’s social position). These conceptualizations of digital abilities reveal the broad array of knowledge, attitudes, and skills involved in ICT.

Based on our consultation with teachers during the pandemic, the sudden shift to online teaching brought about by the school closures meant that some digital skills proved to be critical, such as the ability to communicate using email and messaging applications, the ability to manage a website, use digital office suites to edit documents, create educational videos and/or to stream video sessions followed by uploading their recordings, and use distance learning platforms. Due to their immediate practical value demonstrated during the rapid migration to teaching online which took place in 2020, we use these specific tasks as a basis for measuring self-assessed digital teaching competences.

### The Relationship Between Actual Digital Competences and Perceived Digital Competences

Subjective self-assessment can be defined as an estimation of how skilled or competent an individual is regarding a particular skill, ability, or characteristic (Maderick et al., 2016); therefore, in our field, perceived digital competences is an estimation of how digitally skilled or competent teachers are regarding a particular digital skill, ability, or characteristic. This concept correlates with that of digital self-efficacy, building on Bandura (1994) more general conceptualization, which defines self-efficacy as people’s beliefs about their abilities to perform in activities that influence their lives and, thus, is a capacity that determines how they feel, think, motivate themselves, and behave. Therefore, even if these beliefs do not directly determine whether or not success will be achieved in practice, they still have an influence that affects what individuals choose to do, how they do it, and thus indirectly whether or not they have a chance at succeeding in a particular task (Guo et al., 2015; Brickman, 2021). Wong et al. (2021) in their research on secondary school teachers’ psychological status and competences in E-teaching during COVID-19, showed a negative relationship between psychological status and e-teaching competences (−0.286, *p* < 0.01), as well as reported a negative relationship between the dimension of psychological status and competences in e-teaching.

“Computer anxiety (ICT anxiety) is a generalized emotion of uneasiness, apprehension, the anxiousness of coping, or distress in anticipation of negative outcomes from computer-related operations” (Chang, 2005, p. 715). It is “the feeling of discomfort when using computers (technology more broadly in the context of our study)” (Awofala et al., 2017, p. 92). Indeed, teachers’ ICT anxiety is one of the main obstacles to integrating ICT into their education practice (Saxena et al., 2019). In the context of professionals, researchers have found that computer anxiety has a strong negative effect on computer-related activities, such as computer skills, intention to use computers, attitudes toward computers, and perceived usefulness of computers/ICT (Aktag, 2015; Awofala et al., 2017, 2019). Furthermore, Awofala et al. (2019) found that computer anxiety correlates negatively with self-efficacy.

In certain contexts, perceived digital competences can prove to be a rather valid indicator of actual digital skills (Porat et al., 2018). It does not replace actual behaviors and activities measured in objective terms but captures information on issues and events of the aspect of reality under investigation that could not otherwise be obtained (Mazziotta and Pareto, 2012). Furthermore, self-assessments may depend not only on the objective situation (in this case the actual digital skills possessed by Romanian teachers) but also on the reporting style, which may lead to erroneous conclusions about the actual digital literacy of respondents (Černochová et al., 2020). Nevertheless, researchers show that, regardless of the skills/competences assessed, individuals tend to overestimate their abilities. Maderick et al.’s (2016) review shows that the expertise on the material being tested; the level of difficulty of the material; the specificity of the ability being evaluated; the desirability of the particular skill or ability; gender differences; possible cultural differences; and individual differences in ability are the main factors that lead to erroneous estimation of one’s abilities. It has also been suggested that individuals with low levels of expertise or training will tend to overestimate their knowledge and skills in their given domain, simply because they are unaware of their level of competence (Maderick et al., 2016). This may apply to digital competences as well: Individuals who do not have or do not know the level of their digital knowledge and skills may tend to overestimate or underestimate the level of their digital skills. In contrast, Pavić and Černja (2019) found that those who have a low level of digital skills are aware of this and do not exaggerate their self-assessment as much as those who are aware of possessing a higher level of digital skills. This comparative lack of false claims is possibly due to a high level of motivation and dedication to learning how to improve. Therefore, teachers must correctly measure their digital skills to know the level from which they start in online teaching, but currently, there is no known method by which this would be possible in Romania.

However, in existing research, any form of subjective self-assessment, when compared with more objective methods, tends to demonstrate some degree of inaccuracy (Maderick et al., 2016); however, if considered in conjunction with other, more objective means, self-assessment may prove to be useful for teachers in reflecting upon their competence, skills, and knowledge, therefore aiding them in adjusting their perceptions and attitudes regarding technology throughout their professional practice (Huang and Liang, 2015; Maderick et al., 2016). For example, in Khokhar and Javaid’s (2016) study, while the surveyed teachers believed that they were using ICT effectively, their students reported that their ICT use was not creative or innovative, and instead wanted them to create authentic teaching and learning classroom experiences. These Students’ wishes are an important source of guidance as it has been found that their perceived digital competence and attitudes toward using digital technologies significantly and positively influence their engagement in the learning process (Huang and Liang, 2015).

By way of a conclusion, as Shrauger and Osberg (1981) said, it is unwise to assume that individuals can accurately assess their skills and abilities because they are fundamentally unaware of these capacities on an objective level and tend to present themselves in what they consider to be a socially desirable way. Therefore, in the context of this paper, the concept of “perceived digital competences” refers largely, but not entirely, to the actual digital skills possessed by Romanian teachers as the core of the effective ICT performance required to ensure the positive impact of digital teaching.

### Indicators of Teachers’ Digital Competences

There is no bounty of literature on either the predictors of teachers’ ICT skills or on their self-perception of these skills. According to literature reviews, teachers’ acquisition of skills for use in online environments are conditioned by infrastructure; ICT devices available in the school; training in digital applications; cognitive skills and socio-emotional skills (Bacter et al., 2021), “supported by effective lifelong learning systems” OECD, 2019b,c,d); school environment; academic engagement; and appropriate ongoing technical support (Hatos, 2019; Akmal et al., 2021). The majority of these predictors refer to the contextual and individual resources available to teachers; however, age and gender are two additional well-known predictors of digital skills.

#### Age

At first glance, it may be assumed that younger teachers will have a higher score regarding perceived digital competences than older teachers and that older teachers will have lower actual ICT competences. Indeed, Fernandez-Cruz and Fernandez-Diaz (2016) found that older teachers (i.e., 56–66 years old) with extensive teaching experience have a much lower ICT competence profile than teachers who are younger and have less experience; they also found that teachers aged between 20 and 25 have the best ICT competence profile. No significant effect of teachers’ self-assessed digital skills and age was found by other researchers as well (Drossel et al., 2017; Gil-Flores et al., 2017). However, other studies find that the use of ICT is not influenced by the teacher’s age, but by their number of years in service. In Gu et al. (2013), teachers with less than 5 years of teaching experience were found to use technology less than those with a longer service period.

Research indicates that age might not be a determining factor for digital competences but may contribute to the impact of other age-related circumstantial factors (Law et al., 2008; Majumdar, 2015). However, more recent research has identified a negative relationship between age and ICT use (Juhaňák et al., 2019), or found that digital competence decreases with age (Sundqvist et al., 2020). This idea we intend to carry forward in our study is due to the gap between digital natives and digital immigrants highlighted by researchers (Zenios and Ioannou, 2018; Kesharwani, 2020).

1. H1: Teachers’ self-assessed digital skills are negatively correlated with age.

#### Gender

Several studies have suggested that male teachers tend to view themselves (perceived digital skills) as more technologically adept and willing to learn about new technology, compared to their female counterparts (Cruz and Díaz, 2016; Ghavifekr et al., 2016; Orser and Riding, 2018). Drossel et al. (2017) reported that a teacher’s gender has a significant effect on the frequency of their computer use in classroom settings in three out of the five selected countries: they found that female teachers use computers for instructional purposes more frequently than male teachers in the Netherlands, and male teachers use computers in classroom settings more frequently than female teachers in Poland and Germany. Therefore, it is not yet conclusive whether gender plays a significant role in shaping teachers’ ICT use.

1. H2: male teachers evaluate their digital skills more positively than female teachers.

#### Context (Rural Schools vs. Urban Schools)

There are few studies on teachers’ perceptions of their ICT skills concerning their location (urban vs. rural), but we can assume that there are differences in their perceived ICT skills if we consider other indicators from previous studies (infrastructure, GDP per capita, quality of education, other social opportunities). For example, Koen et al. (2017) show that there are persistent and growing differences in data infrastructure between urban and rural areas and, if we view urban areas as having higher socioeconomic status, this is an indirect, positive correlation with the availability of ICT resources (Yang et al., 2019). Furthermore, given that teachers in urban schools have been found to use ICT more frequently than those in semi-urban schools (Buabeng-Andoh, 2019), perhaps we can assume that the frequency is higher than in rural areas as well. According to the same study, urban school teachers receive more training and leadership support than those in semi-urban schools (Buabeng-Andoh, 2019), and, again, perhaps we can expect that it is also more than rural school teachers receive. Hamzah et al. (2021) ’s results about the effects of principals’ digital leadership on teachers’ digital teaching during the COVID-19 pandemic in Malaysia indicate that the level of digital leadership displayed by principals and teachers’ digital teaching practice are in a positive correlation and that the ability to plan and organize digital leadership programs is important and can help improve Students’ academic performance, despite the COVID-19 pandemic crisis (Karakose et al., 2021a). The last reason for our hypothesis is that urban teachers have been reported to be less anxious about using ICT in the classroom than those in rural areas, a difference found to be especially pronounced among female teachers (Saxena, 2014; Saxena et al., 2019).

1. H3: Teachers in urban schools evaluate their ICT skills more positively than teachers in rural schools.

#### Teachers’ Qualifications and Status in the Teaching Profession

Fernandez-Cruz and Fernandez-Diaz’s (2016) study of the Madrid teaching community (*n* = 1,433 teachers) reports that teachers working in secondary education have better ICT competences than those teaching in primary education. Additionally, Law et al. (2008) and Celik and Yesilyurt (2013) suggest that the highest percentage of ICT-using teachers are generally more likely to be found among those who have obtained the highest qualification category themselves (i.e., master’s degree or above). Moreover, there is evidence that perceived pedagogical competence enhances the use of ICT in pedagogical settings. Furthermore, teachers’ self-perceived competence correlates with a higher mean for general ICT use than for pedagogical ICT-use might indicate that teachers are generally more confident about using ICT in everyday situations than in teaching and learning situations (Fu, 2013). Therefore, we want to know whether teachers with high professional status indicators will assess their digital skills more positively than their colleagues, or not.

1. *H4: teachers with high professional status indicators evaluate their digital skills more positively than their colleagues with lower professional certificates, those working in primary schools or secondary schools, or those with a lower professional status.*

#### The Teacher’s Subject Field

There is very little research on the subject field taught as a predictor of teachers’ perceived digital skills, although it determines the cognitive and motivational resources required for ICT use, as well as opportunities to practice ICT skills (Sundqvist et al., 2020). However, some studies have revealed that teachers of artistic and practical subjects are less skilled than other subject teachers in using digital teaching materials (Tanhua-Piiroinen et al., 2016; Rucsanda et al., 2021), and that science and technology teachers display higher levels of digital competences (Fernandez-Cruz and Fernandez-Diaz, 2016). However, Al Darayseh (2020) ’s study about the impact of the COVID-19 pandemic on modes of teaching science in UAE schools showed that the main challenges for science teachers during the COVID-19 was the absence of hands-on activities, conducting experiments in wet labs, fostering interaction in the online classroom, and managing Students’ behavior. Nonetheless, some studies have shown that students living in countries where teachers with more advanced ICT skills are prevalent tend to perform better than their counterparts in a broad range of subjects: i.e., mathematics, reading, and science (Hu et al., 2018); however, there is no clear evidence between the subject field and teachers perceived digital skills. We expect that teachers in computing and informatics evaluate their ICT skills more positively than other teachers due to the time spent on a computer and the specificity of their activity.

1. *H5: teachers, who are active primarily in STEM fields, particularly in computing and informatics, evaluate their ICT skills more positively than other teachers.*

#### Impact of Previous Training Courses

The existing evidence on the actual impact of training in ICT skills for teachers is scarce. Law et al.’s (2008) research compared the percentages of teachers who had attended two kinds of professional development activities: ICT and pedagogy-related. In their findings, the percentages were higher for technical activities than for pedagogical activities (Law et al., 2008). However, a technical course is not enough for teachers to learn how to integrate ICT into their teaching process (Meyer and Bo-Kristensen, 2012). A significant deficit in teacher training in the use of ICT and its application in the classroom is also revealed by Fernandez-Cruz and Fernandez-Diaz (2016) who show that teachers’ classroom strategies regarding the use of ICT resources as an avenue for complex and collaborative learning have not yet been implemented as teaching methods in the development of Students’ digital competence. However, teachers who had a computer and Internet access at home considered ICT as improving the teaching-learning process had a good level of training in ICTs (Ghavifekr et al., 2014; Ghavifekr and Rosdy, 2015). Despite the general shortage of evidence on this topic, it is reasonable to expect that teachers who have previously attended training in online teaching will evaluate their skills more positively than those who have not.

1. *H6: teachers who have previously attended training in online teaching will evaluate their skills more positively than those without such training.*

As described above, Romania is not geographically homogenous. Therefore, in addition to the stated hypotheses, going forward we introduce county indicators as controls of probable contextual variation in digital resources.

## Methodology

### Data

The data used in these analyses were collected through simple random technique from teachers active in pre-tertiary education in Romania through an online survey conducted by the University of Oradea and the University of Suceava. 3,419 valid self-administered online questionnaires were completed between the 1st and 7th of April 2020. From a territorial point of view, the sample is not representative, as the population of teachers from the NE areas of Romania is overrepresented. In the subsequent analyses, we have used a weighted database to assure representativity regarding the proportions of teachers by the educational cycles and the type of locality in which they teach. Weighting was performed using data about the demographics of Romania’s teaching staff published by the National Institute of Statistics—Institutul Naţional de Statistică (INS, 2018).

### Dependent Variables

Teachers’ perceived digital competences.

Eight Likert-type items of perceived ability were used initially to measure the teachers’ self-assessed digital competences.

1. Creating and editing documents in an Office program (e.g., Word or PowerPoint)
2. Creating and editing educational video content
3. Distributing content, tasks, and feedback to students using email
4. Distributing content using messaging applications (e.g., WhatsApp, Facebook, and Messenger)
5. Organizing video conferences using an appropriate platform (e.g., Zoom, WebEx, and Skype)
6. Creating a videoconference using a video streaming solution (e.g., YouTube, Facebook)
7. Distributing content and tasks, as well as delivering feedback through e-learning or distance learning platforms (e.g., Google Classroom, Moodle, and Microsoft Teams)
8. Developing and managing their website to deliver content

Factor analysis of the teachers’ responses using principal axis factoring and Varimax rotation (KMO = 0.869) revealed the two-dimensional structure of the data, with the first factor covering 53.5% of the variance in the data and the second showing 16.94%. The most important factor is after rotation loadings larger than 0.5 for the items related to video editing; video streaming; videoconferencing; using e-learning and distance learning platforms, and developing and managing websites (items 2, and 5–8). The second dimension covers the items concerning the skills for using digital office programs, emails, and chat and messaging applications (items 1, 3, and 4). Noticeably, the first factor refers to multimedia and online skills, while the second refers to digital office skills: therefore, we will call them Self-assessed Multimedia and Online Skills Score (SMOS) and Self-Assessed Digital Office Skills Score (SDOS), respectively. To preserve as much information as possible from the data, we have used the factor scores as separate measures of the two constructs instead of using cumulative scores as is often the norm in this type of measurement. For both respective scales, alpha is > 0.7.

Since both measures of self-perceived digital skills are factor scores, their average is null, and the standard deviation is close to 1. However, both variables have distributions skewed to the right, with longer tails on the negative sides.

Our scale is a self-assessment tool as are the ones developed by the Digital Skills Accelerator of the EU for example (Misheva, 2021), or the digital competences self-assessment tool of DigiCompEdu (Digital Skills Accelerator, n.d.; Redecker and Punie, 2017), another instrument based on the EU’s digital competences framework. However, ours is a much shorter and simpler one with items tailored to the immediate technical needs of online teaching at the start of the pandemic and the abrupt and masse entrance in online teaching.

### Independent Variables

All independent variables used in the analysis were dummy coded. This was imposed according to their categorical measurement in the survey, including age which was recorded using intervals. The list of independent variables and their distributions are in Table 1.

**TABLE 1:** Independent variables in hierarchical linear regression.

**Dimension**	**Sub-dimension**	**Name of variable**	**Measurement**	**0**	**1**	**% yes**
Gender	Gender	Female01	Dummy (1 = female)	590	2,805	82.6
Age	Age	Age: yrs31_40	Dummy	2,474	920	27.1
		Age: under30	Dummy	3,149	246	7.2
		Age: yrs41_50	Dummy	2,020	1,375	40.5
		Age: yrs51_60	Dummy	2,670	724	21.3
		Age: over60	Dummy	3,265	129	3.8
Context of school	Place of school	Place: urban_school	Dummy	1,299	2,096	61.7
	Domicile of teacher	Domicile: same place with school	Dummy	1,325	2,069	61.0
	County	County: Arad	Dummy	3,274	121	3.6
		County: Bihor	Dummy	3,100	295	8.7
		County: Botoşani	Dummy	2,712	683	20.1
		County: Covasna	Dummy	3,237	158	4.7
		County: Maramureş	Dummy	3,197	198	5.8
		County: Neamţ	Dummy	2,289	1,105	32.6
		County: Suceava	Dummy	2,841	554	16.3
Status in the profession	Employment status	Employment status: replacement	Dummy	2,985	409	12.1
		Employment status: tenure	Dummy	478	2,916	85.9
		Employment status: tenure_temporary	Dummy	3,325	69	2.0
Status in the profession	Type of school	Type of school: HS_college_nat	Dummy	3,131	263	7.7
		Type of school: HS_college_tech	Dummy	3,128	266	7.8
		Type of school: HS_lic_technological	Dummy	2,987	407	12,0
		Type of school: HS_lic_theor	Dummy	3,198	196	5.8
		Type of school: HS_lic_vocational	Dummy	3,194	201	5.9
		Type of school: Lower_secondary_school	Dummy	1,491	1,904	56.1
		Type of school: Primary_school	Dummy	3,237	157	4.6
Status in the profession	Teacher’s degree	Degree: definitive	Dummy	2,893	502	14.8
		Degree: doctor	Dummy	3,297	98	2.9
		Degree: degree_grade1	Dummy	1,216	2,178	64.2
		Degree: degree_grade2	Dummy	2,919	475	14.0
Field	Field	Field: Humanities languages, history, religion, arts	Dummy	2,152	1,242	36.6
		Field: sciences_ch_ph_bio Chemistry, physics, biology	Dummy	3,064	330	9.7
		Field: Mathematics	Dummy	3,076	318	9.4
		Field: ITC_informatics	Dummy	3,234	161	4.7
Attended digital teaching courses	Attended digital teaching courses	Attended digital teaching courses	Dummy	2,968	426	12.6

### Analytic Strategy

The stated hypotheses were tested using hierarchical linear regressions of the two independent variables separately. To achieve the purpose of identifying interactions and mediation effects, we have grouped the independent variables into eight blocks that were added sequentially to the regression models. Not all dummies covering a dimension were introduced in modeling due to redundancy (the number of dummies included in regression must be mostly *n* = 1 of the number of categories of the original categorical variables) and because of collinearities. Furthermore, given the almost orthogonal relationship between the two dependent variables (correlation), it is likely that their sources of variation are somewhat different (see Table 2).

**TABLE 2:** The sequence of variables included in hierarchical linear modeling.

**Block**	**Dimensions, variables**
1	Gender, Age (under 30, 31–40, 41–50, over 60)
2	Place of school (urban vs. rural), Domicile of teacher (same place with school)
3	County (Arad, Bihor, Botoşani, Covasna, Maramures, Neamt, Suceava)
4	Employment status (replacement or temporary tenure)
5	Type of school (National college, Technical college, Technological lyceum, Lower secondary school, Primary school)
6	Teacher’s degree (definitive, doctor—phd, grade 2)
7	Field (humanities, sciences, mathematics, IT)
8	Attended digital teaching courses

## Results

### Hierarchical Linear Regressions: Model Fit Change

The final explanatory power of the regressions for both dependent variables slightly exceeds 7.5% of the total variances, with a positive value for SDOS, where 8.2% of the variance is covered by the 29 dummy variables included in the final model (Table 3). Such a low explanatory power is due to the large size of the sample, the absence of some important predictors from the specifications of the models, e.g., home ICT resources and ICT experience at home and outside of school, and the penalty incurred by many predictors when computing adjusted *R*^2^.

**TABLE 3:** Model fit change in hierarchical linear modeling.

	**SMOS**			**SDOS**		
**Model**	**Adjusted R square**	**df1**	**Sig. F change**	**Adjusted R square**	**df1**	**Sig. F change**
1	0.030	5	0.000	0.028	5	0.000
2	0.031	2	0.079	0.052	2	0.000
3	0.040	7	0.000	0.055	7	0.014
4	0.040	2	0.083	0.057	2	0.003
5	0.041	5	0.270	0.062	5	0.001
6	0.041	3	0.191	0.062	3	0.106
7	0.059	4	0.000	0.074	4	0.000
8	0.076	1	0.000	0.082	1	0.000

Our specifications more effectively cover the variation of the Self-Assessed Digital Office Skills Score (SDOS) than the Self-Assessed Multimedia and Online Skills Score (SMOS).

While variables indicating teachers’, professional certificates do not contribute at all to the variance of the dependent variables, as three other blocks have a significant impact only on SDOS, i.e., location of school; the domicile of teacher compared with the location of school and employment status of teacher and type of school employing a teacher. The variables in the remaining three blocks have significant parameters: gender and age, the field in which the teacher is teaching, and whether online teaching courses were attended.

### Models Compared

The detailed tables of the parameters of hierarchical regressions reveal interesting differences between the two scores of self-perceived skills as much as suggesting the impact of status in the profession on SDOS (Table 4).

**TABLE 4:** The models compared.

		**SMOS**		**SDOS**	
**Model**		**Beta**	**Sig**	**Beta**	**sig**
1	(Constant)		0.538		0.000
	Female01	−0.101	0.000	0.062	0.000
	Age: under30	0.128	0.000	0.117	0.000
	Age: yrs31_40	0.126	0.000	0.115	0.000
	Age: yrs41_50	0.099	0.000	0.119	0.000
	Age: over60	−0.033	0.066	−0.057	0.002
2	(Constant)		0.678		0.000
	Female01	−0.101	0.000	0.061	0.000
	Age: under30	0.134	0.000	0.141	0.000
	Age: yrs31_40	0.132	0.000	0.137	0.000
	Age: yrs41_50	0.101	0.000	0.126	0.000
	Age: over60	−0.033	0.072	−0.054	0.002
	Place: urban school	0.036	0.056	0.150	0.000
	Domicile: same place with school	0.004	0.819	0.018	0.350
3	(Constant)		0.767		0.000
	Female01	−0.102	0.000	0.059	0.000
	Age: under30	0.139	0.000	0.143	0.000
	Age: yrs31_40	0.133	0.000	0.140	0.000
	Age: yrs41_50	0.097	0.000	0.124	0.000
	Age: over60	−0.034	0.063	−0.055	0.002
	Place: urban school	0.037	0.054	0.145	0.000
	Domicile: same place with school	0.009	0.659	0.014	0.473
	County: Arad	−0.027	0.171	0.010	0.603
	County: Bihor	−0.029	0.209	−0.049	0.033
	County: Botoşani	−0.043	0.123	−0.041	0.144
	County: Covasna	−0.002	0.923	−0.005	0.806
	County: Maramureş	0.025	0.233	0.024	0.265
	County: Neamţ	0.019	0.547	−0.062	0.043
	County: Suceava	−0.084	0.002	−0.023	0.378
4	(Constant)		0.850		0.000
	Female01	−0.101	0.000	0.058	0.001
	Age: under30	0.125	0.000	0.167	0.000
	Age: yrs31_40	0.127	0.000	0.151	0.000
	Age: yrs41_50	0.096	0.000	0.125	0.000
	Age: over60	−0.035	0.056	−0.053	0.003
	Place: urban school	0.038	0.046	0.141	0.000
	Domicile: same place with school	0.011	0.563	0.008	0.685
	County: Arad	−0.028	0.164	0.010	0.606
	County: Bihor	−0.031	0.185	−0.046	0.046
	County: Botoşani	−0.044	0.115	−0.039	0.156
	County: Covasna	−0.002	0.918	−0.005	0.824
	County: Maramureş	0.025	0.239	0.024	0.254
	County: Neamţ	0.017	0.586	−0.059	0.054
	County: Suceava	−0.083	0.002	−0.024	0.358
	Employment status: replacement	0.025	0.170	−0.059	0.001
	Employment status: tenure_temporary	0.033	0.055	−0.025	0.141
5	(Constant)		0.977		0.001
	Female01	−0.101	0.000	0.064	0.000
	Age: under30	0.123	0.000	0.174	0.000
	Age: yrs31_40	0.126	0.000	0.155	0.000
	Age: yrs41_50	0.096	0.000	0.126	0.000
	Age: over60	−0.034	0.062	−0.050	0.005
	Place: urban school	0.047	0.026	0.106	0.000
	Domicile: same place with school	0.011	0.568	0.004	0.834
	County: Arad	−0.029	0.138	0.011	0.574
	County: Bihor	−0.032	0.173	−0.049	0.034
	County: Botoşani	−0.043	0.125	−0.032	0.247
	County: Covasna	−0.004	0.848	−0.005	0.812
	County: Maramureş	0.029	0.179	0.027	0.208
	County: Neamţ	0.028	0.371	−0.056	0.069
	County: Suceava	−0.079	0.003	−0.018	0.505
	Employment status: replacement	0.027	0.148	−0.062	0.001
	Employment status: tenure_temporary	0.034	0.045	−0.026	0.123
	Type of school: HS_college_nat	0.012	0.572	0.027	0.197
	Type of school: Lower_secondary_school	−0.001	0.973	−0.073	0.011
	Type of school: Primary_school	0.012	0.544	−0.054	0.008
	Type of school: HS_college_tech	−0.04	0.065	−0.007	0.744
	Type of school: HS_lic_technological	−0.004	0.868	−0.026	0.258
6	(Constant)		0.871		0.001
	Female01	−0.102	0.000	0.065	0.000
	Age: under30	0.121	0.000	0.182	0.000
	Age: yrs31_40	0.13	0.000	0.167	0.000
	Age: yrs41_50	0.098	0.000	0.129	0.000
	Age: over60	−0.033	0.067	−0.049	0.006
	Place: urban school	0.049	0.021	0.105	0.000
	Domicile: same place with school	0.011	0.561	0.001	0.953
	County: Arad	−0.031	0.119	0.011	0.580
	County: Bihor	−0.032	0.171	−0.049	0.033
	County: Botoşani	−0.044	0.117	−0.029	0.289
	County: Covasna	−0.004	0.849	−0.002	0.904
	County: Maramureş	0.029	0.181	0.026	0.217
	County: Neamţ	0.026	0.401	−0.055	0.073
	County: Suceava	−0.079	0.003	−0.018	0.507
	Employment status: replacement	0.023	0.219	−0.059	0.002
	Employment status: tenure_temporary	0.033	0.058	−0.024	0.157
	Type of school: HS_college_nat	0.013	0.545	0.024	0.249
	Type of school: Lower_secondary_school	−0.004	0.888	−0.071	0.013
	Type of school: Primary_school	0.011	0.586	−0.052	0.009
	Type of school: HS_college_tech	−0.041	0.058	−0.007	0.754
	Type of school: HS_lic_technological	−0.005	0.843	−0.025	0.275
	Degree: definitive	0.008	0.679	−0.020	0.297
	Degree: doctor	−0.032	0.064	0.026	0.122
	Degree: grade2	−0.018	0.318	−0.030	0.098
7	(Constant)		0.642		0.000
	Female01	−0.095	0.000	0.074	0.000
	Age: under30	0.121	0.000	0.188	0.000
	Age: yrs31_40	0.135	0.000	0.173	0.000
	Age: yrs41_50	0.099	0.000	0.133	0.000
	Age: over60	−0.03	0.095	−0.045	0.012
	Place: urban school	0.043	0.042	0.107	0.000
	Domicile: same place with school	0.011	0.565	0.004	0.840
	County: Arad	−0.033	0.098	0.017	0.379
	County: Bihor	−0.032	0.170	−0.044	0.056
	County: Botoşani	−0.044	0.118	−0.028	0.305
	County: Covasna	−0.002	0.937	−0.001	0.979
	County: Maramureş	0.023	0.288	0.018	0.384
	County: Neamţ	0.029	0.349	−0.050	0.105
	County: Suceava	−0.081	0.002	−0.021	0.419
	Employment status: replacement	0.02	0.281	−0.067	0.000
	Employment status: tenure_temporary	0.03	0.083	−0.023	0.184
	Type of school: HS_college_nat	0.013	0.534	0.019	0.355
	Type of school: Lower_secondary_school	−0.002	0.953	−0.065	0.022
	Type of school: Primary_school	0.002	0.928	−0.038	0.064
	Type of school: HS_college_tech	−0.038	0.074	−0.001	0.960
	Type of school: HS_lic_technological	−0.007	0.767	−0.024	0.285
	Degree: definitive	0.007	0.739	−0.023	0.230
	Degree: doctor	−0.023	0.184	0.026	0.117
	Degree: grade2	−0.018	0.304	−0.032	0.076
	Field: Humanities Languages, history, religion, arts	−0.052	0.005	0.038	0.039
	Field: sciences_ch_ph_bio Chemistry, physics, biology	−0.058	0.001	0.018	0.311
	Field: Mathematics	−0.038	0.027	0.023	0.174
	Field: ITC_informatics	0.118	0.000	0.112	0.000
8	(Constant)		0.988		0.000
	Female01	−0.097	0.000	0.072	0.000
	Age: under30	0.124	0.000	0.190	0.000
	Age: yrs31_40	0.138	0.000	0.175	0.000
	Age: yrs41_50	0.102	0.000	0.134	0.000
	Age: over60	−0.026	0.141	−0.042	0.017
	Place: urban school	0.04	0.055	0.105	0.000
	Domicile: same place with school	0.01	0.594	0.003	0.865
	County: Arad	−0.031	0.114	0.018	0.346
	County: Bihor	−0.029	0.213	−0.042	0.067
	County: Botoşani	−0.045	0.100	−0.030	0.283
	County: Covasna	0	0.998	0.000	0.980
	County: Maramureş	0.018	0.386	0.015	0.461
	County: Neamţ	0.031	0.308	−0.048	0.114
	County: Suceava	−0.086	0.001	−0.024	0.350
	Employment status: replacement	0.026	0.164	−0.063	0.001
	Employment status: tenure_temporary	0.029	0.084	−0.023	0.177
	Type of school: HS_college_nat	0.009	0.663	0.017	0.424
	Type of school: Lower_secondary_school	0	0.991	−0.064	0.024
	Type of school: Primary_school	0.003	0.902	−0.037	0.066
	Type of school: HS_college_tech	−0.038	0.076	−0.001	0.975
	Type of school: HS_lic_technological	−0.003	0.899	−0.022	0.338
	Degree: definitive	0.005	0.780	−0.024	0.214
	Degree: doctor	−0.025	0.144	0.025	0.136
	Degree: grade2	−0.015	0.393	−0.029	0.097
	Field: Humanities Languages, history, religion, arts	−0.052	0.005	0.038	0.041
	Field: sciences_ch_ph_bio Chemistry, physics, biology	−0.057	0.001	0.018	0.290
	Field: Mathematics	−0.039	0.024	0.023	0.180
	Field: ITC_informatics	0.110	0.000	0.107	0.000
	Attended digital teaching courses	0.130	0.000	0.086	0.000

Stepwise comparisons of the parameters in the block models do not reveal significant interactions and mediation effects with salient suppression effect of status in the profession on the impact of age. The most salient difference between the models for the two dependent variables is the contrast found regarding teachers’ genders. Female teachers reported perceiving their multimedia and online digital skills at a lower level than their male counterparts. As such, it seems that female teachers view themselves as specializing in solving digital office tasks more than male teachers, while male teachers view themselves to be more skilled in the creative roles of devising and distributing online and multimedia content.

In terms of the relationship between the teachers’ ages and their self-assessed digital competences, for both dependent variables, belonging to the oldest age group correlates with a lower reported competence level in contrast with the younger ones. However, there are some differences between the two dependent variables: while age group parameters remain almost unchanged across models for SMOS, they increase in the case of SDOS with every group of variables added. This suggests that where the variation of Self-Perceived Digital Office Skills is concerned, the impact of age is suppressed by other variables, especially by those in the second block (i.e., location of work and domicile), those in the fourth block (i.e., employment status), and teachers’ degrees. In simple terms, this suppression is since positions that indicate higher status in the education field (e.g., tenure, grade, or being a teacher in urban areas) suppose better digital office skills, but simultaneously are negatively related to age. Controlling for status in the education field makes the impact of age on digital office skills more evident. The impact of age group on SDOS is the largest out of all the regressions if the size of betas is considered.

Regarding the relationship between the geographical location of schools and teachers’ self-assessed digital competences, teachers working in urban schools report having higher SMOS and SDOS than their rural counterparts, with the parameters being larger in the case of digital office skills. On the other hand, both the teacher being a resident in the same place as the school where they teach or having to commute are non-consequential in terms of their influence on the dependent variable.

With the notable exception of Suceava, counties in which the teachers teach generally do not have significant parameters on either dependent variable. Teachers residing in counties in NE Romania, mainly Suceava, and with smaller parameters in Botoşani and Neamţ, appear to have a lower self-assessment of their digital, online, and multimedia skills.

Regarding the influence of professional status on self-assessed digital competences, the blocks that included variables designating the status of subjects in the profession (i.e., employment status and type of school) have a significant positive impact (i.e., the higher the status, the larger the dependent measure) in the case of self-assessed digital office skills. Conversely, replacement teachers and those in lower secondary schools (i.e., gymnasiums) evaluate their digital office skills especially poorly.

Excluding the impact of age, the (positive) effects of being an ICT teacher and of having attended a distant education training, respectively, are the strongest for both dependent variables. It is interesting to note here that SMOS is significantly smaller for teachers of all other topics included in the modeling, which is not the case for SDOS.

## Discussion

Multivariate modeling of the two measures of self-perceived digital skills produced some rather straightforward results, although the interpretation of these results is not necessarily undoubtful.

### Self-Assessed Digital Skills and Age Relationship

In the findings of the present study, age plays an expected role in teachers’ perception of their digital skills. Socialized and accustomed to digital devices, systems, and environments, younger teachers have more confidence in their ICT skills regardless of other characteristics. These results are like those of Fernandez-Cruz and Fernandez-Diaz (2016), who also found that self-assessed digital skills are negatively correlated with age for teachers.

However, the most interesting results are those that relate SMOS and SDOS with gender, age, and status in the profession, thus revealing some of the internal dynamics of the teaching profession and its relationship with digital skills.

### Self-Assessed Digital Skills and Gender Relationship

The contrasting parameters of gender in the present study reveal the gendered nature of tasks in the teaching profession, or at least the perceived gender stereotypes concerning these abilities: multimedia and online skills are self-reported as a specialty of male teachers, while digital office skills are self-reported as a specialty of female teachers. Indeed, it is possible to speculate more broadly here around the gendered nature of work roles and workplace hierarchies, or indeed about the recent history of women being forced into clerical or secretarial roles (Alshabani et al., 2020; Balka and Wagner, 2020). However, several studies show that there is no significant effect of gender on actual digital skills, and instead, differences in skill sets are more likely to relate to social, historical, cultural, or other contextual differences between male and female teachers (Law et al., 2008). The difference of perceived digital skills in favor of male teachers in the present study may also be caused by narcissistic aspects of their personality (Philipson, 1985), leading them to self-assess their ICT skills more highly than their actual ICT skills.

### Self-Assessed Digital Skills and School Location Relationship

Teachers in urban schools have higher perceived digital skills, with the parameters being larger in the case of digital office skills. Teachers being resident in the same place as the school where they teach or having to commute are both non-consequential upon the dependent variable. Indeed, this result is similar to Koen et al. (2017); however, taking into consideration the socioeconomic status of the areas in which the schools are located as well, it is perhaps almost self-evident that the urban school teachers will have greater perceived digital skills, given that urban areas are recipient to greater investment, infrastructure, and training opportunities (Wiesel and Liu, 2020). Eroğlu and Şenol (2021) made phenomenological research about emergency remote education experiences of teachers during the COVID-19 Pandemic with students being mostly in the low socioeconomic group. Their results show that emergency remote education was ineffective due to low student participation, insufficient infrastructure, lack of responsibility and motivation for learning, low ICT competency of students and teachers, low socioeconomic status, and inappropriateness of planning and curriculums.

### Self-Assessed Digital Skills and Professional Status Relationship

The parameters revealing the fact that teachers occupying peripheral positions in the teaching professions are equally intriguing (i.e., replacement teachers and those teaching in lower-secondary schools or primary schools) as lower self-assessed office digital skills than those with positions was demonstrated as more important; on the other hand, the same trend does not exist in the case of multimedia and online skills. Evidently, ascension in the professional hierarchy can be partially facilitated by having digital office skills, and the reverse causation could also be possible; however, this is a self-perpetuating truth as the higher the standing of the teacher, the more they have the opportunity to practice digital skills for certain tasks, on the principle of the Matthew effect: “the one who has it will be given to him” (Mingo and Bracciale, 2018). Furthermore, researchers suggest that higher qualifications lead to teachers developing and having higher levels of actual digital skills, and this might be the consequence of the combination of proper knowledge, skills, and attitudes that increase the innovative use of ICT (Gudmundsdottir and Hatlevik, 2018; West et al., 2019).

### Self-Assessed Digital Skills and School Subject Relationship

In the present study, it was also expected that specialization in IT, i.e., as a teacher of ITC and informatics—would significantly predict higher SDOS and SMOS. However, the fact that other subject fields appear to have a negative impact on SMOS, or no effect at all in the case of SDOS, is perhaps less intuitive.

**FIGURE 2:**
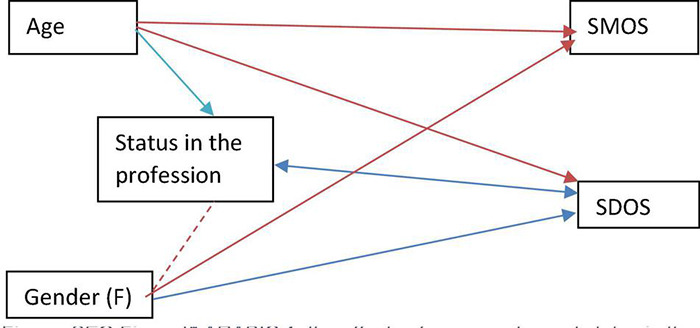
Correlation between factors.

The present study results suggest that any correlation that might be perceived between the subject field and self-assessed digital skills would be erroneous, produced by confounding factors. In this case, gender and status in the field are evident variables whose impact could be confounded with that of the subject field (see Figure 2).

The impact of the technology field is confirmed in other studies as well (Fernandez-Cruz and Fernandez-Diaz, 2016), but an interesting avenue for further research would be to ascertain whether teachers with a high level of self-assessed digital skills report themselves to do better than other teachers at other activities as well (e.g., pedagogical skills).

### Self-Assessed Digital Skills and Professional Training Relationship

In the present study’s results, prior participation in distance teaching training is positively related to the dependent variables which could be understood, on an immediate level, as a simple indicator of the impact of that training. However, on the one hand, this correlation could be partly fallacious, explained instead by teachers’ interest in online teaching, and on the one hand, causality could also be inferred from self-perceived skills to participate in distance education training sessions. There are no relevant results to compare with the present study. Law et al. (2008) only draw attention to the difference between online skills in training courses, by promoting technical and pedagogical skills in an online environment.

Considering the reverse sign effects involving these predictors, regarding the two dependent variables, though statistically not significant, it could be hypothesized that here, as in the case of the effect of gender, there are two contrasting causal mechanisms. Based on the results of the present study, it can be inferred that having multimedia and online skills is not yet a correlate of a successful career in the teaching profession. Further investigations are required, however, to clarify what the actual relation of advancement in career is for teachers with certain digital skills, along with whether or not these relationships are connected with structural variables like age, length of career, and gender, as might seem plausible given the current study results.

### Educational Implications

This critical period, a global pandemic, has been a wake-up call, with schools closed and lessons suddenly transferred online (Lim, 2020). Few teachers were readily able to do this (Edelhauser and Lupu-Dima, 2020), hence their (ongoing) need to attend courses to enhance their skills and abilities in using technology in their professional practice. On the other hand, going forward, schools should consider this need more seriously and invest the necessary funds for equipping laboratories and classrooms with computers, as well as in-service teacher training courses regarding digital tools for education. There is still a significant problem with teacher training in terms of efficiently using technology for various reasons: some teachers either do not want to learn how to use it, the training courses are not effective, or put simply, some of them fundamentally do not agree with using ICT (Holotescu et al., 2020). Indeed, Gudmundsdottir and Hatlevik (2018) highlight recent research that indicates a discrepancy between digital requirements and teachers’ training in technology use. Many teachers perceive themselves to have a low level of computer competence (Santi et al., 2020), thus they develop a neutral attitude toward ICT or they only use technology when it is necessary (Sundqvist et al., 2020), as they may be anxious and unconfident in their use of technology (Klapproth et al., 2020). In this case, teachers can be understood as “digital immigrants” in that they only learn about ICT tools, but do not necessarily immerse or live with a positive attitude about new technologies (Kesharwani, 2020; Anzari et al., 2021; Noronha-Sousa et al., 2022). Education cannot ignore the rapid and far-reaching development of technology and its applications. In practice, this presents a real challenge because ICT is involved across the entire education system: from organizational change (e.g., time and place for independent or collaborative learning, tailored instruction, etc.), to means of delivering educational content (Heitink et al., 2016).

At present, there is a need for comprehensive and up-to-date education policies regarding digital competences, both for educational management (Karakose et al., 2021b) and for teacher training (OECD, 2019e). In terms of pre-service teacher training, it is important for universities to provide quality training and to adapt all courses to address new technologies and trends (Engen and Engen, 2019). As such, it is crucial to have highly qualified university teachers who are developing new methods and tools for teaching and learning. Post-pandemic, modern pedagogy must be fundamentally revised, including elements of e-learning (Paniagua and Istance, 2018): namely regarding the use of digital tools in education and promoting in-service training for teachers (Napal-Fraile et al., 2018).

On an education management level, there are several priorities: adapting all learning processes to fit with new technology, analyzing available resources (i.e., both human and technological), developing ways of communicating *via* the internet (e.g., Facebook, WhatsApp, and multimedia tools), and keeping abreast of new and emerging pedagogical trends for digital teaching and learning (Shonfeld et al., 2020). There is cause for hope, as, in recent years, literature has highlighted that teachers are increasingly becoming followers of technology when they have access to comprehensive infrastructure, ICT devices in schools, training, and support in their school environments and communities (Hatos, 2019). A study about teachers’ perspective on school development at German vocational schools during the COVID-19 pandemic shows that the main coping strategies proved to be a clear agenda by the school leadership in connection with reliable technological infrastructure and teachers’ willingness to use digital teaching methods and König et al. (2020) and Delcker and Ifenthaler (2021) in his study about adapting to online teaching during COVID-19 school closure in the same country, show that information and communication technologies (ICT) tools, particularly digital teacher competence and teacher education opportunities to learn digital competence, are instrumental in adapting to online teaching during COVID-19 school closures. The Ministry of Education can even adopt system and technology innovations that will expand the use of distance learning and distance or alternative assessments (OECD, 2020c).

Ultimately, technology, and teachers’ effective use of it (Al Kodri, 2021), is an invaluable means of creating a learning and working environment for twenty-first-century students (especially during the online schooling period during the COVID-19 pandemic), helping them develop co-operation skills, communication abilities, problem-solving skills, and capacity for continuous learning (Huda et al., 2018), providing teachers with the necessary training and framework for technology-related professional development (Choi et al., 2021), and as Christopoulos and Sprangers (2021) say: simultaneously careful examining the characteristics of proposed platforms or tools and a trial of such characteristics before integration within an educational system.

### Ethical Considerations

Core principles of ethical considerations, for which there was complete agreement across the authors, were consistently adhered to across all participating institutions. The participants gave their free, informed consent, were aware of their right to withdraw from the study, and understood that all data would be anonymized. The participants were all teachers, and they are highly educated adults able to fully understand these concepts and, as such, this was deemed to be a low-risk study by all institutions.

### Limits of the Present Research

The present research faced several limits in achieving its objectives. Firstly, there is a distinct possibility of bias in the sample as it was self-selected using an online questionnaire. Self-selection of the sample, as well as the self-reported technique used, might have correlated with the dependent variables, thus potentially distorting the results.

Another significant issue with the present research is the absence of a measure of actual digital skills. Therefore, it cannot be assessed how much these real skills reflect the teachers’ perception of their digital skills. This translates into another limitation: the underspecified value of the multiple regressions. It is plausible to assume that a large part of the significant unexplained variance is covered by the impact of actual skills, which should have been measured using observational indicators.

## Conclusion

The present research analyzed the perceived digital skills of Romanian pre-tertiary cycle teachers concerning their professional status, the context of the school in which they teach, their gender, their age, their taught field, and their previous participation (or lack of) in training for online teaching. Using data from 3,419 questionnaires in an online survey self-completed by these teachers at the beginning of the COVID-19 lockdown (i.e., the first half of April 2020), two consistent measures of perceived digital skills have been built as factor scores from 8 items, yielding Self-assessed Multimedia and Online Skills Scores (SMOS) and Self-Assessed Digital Office Skills Scores (SDOS), i.e., the dependent variables in this study. Based on current literature on the predictors of digital skills, several hypotheses were built concerning the variations of the two measures of the perceived digital abilities of teachers in the primary and secondary cycles (tested using hierarchical linear regressions).

This study’s first conclusion derives from the low explanatory powers of the regressions with full specifications (adjusted *R*^2^ of 0.076 and 0.082, respectively), which suggests that the single most important predictor of perceived digital skills are actual digital skills, whose measure is absent from the model.

Most of the hypotheses are positive:

– Younger teachers have more confidence in their ICT skills regardless of any other characteristics (H1).
– Teachers in urban schools have higher self-perceived digital skills (H3).
– teachers, who are active primarily in STEM fields, particularly in computing and informatics, evaluate their ICT skills more positively than other teachers (in other subject fields) (H5).
– Prior participation in training for distance teaching is positively related to this study’s dependent variables (H6).

However, exceptions persist, especially concerning H2 and H4:

– Self-perceived multimedia and online skills are self-reported as a specialty of male teachers, while self-perceived digital office skills are self-reported as a specialty of female teachers. It is unclear if this notable contrast mirrors actual skills or is a by-product of gendered stereotypes impacting self-assessment.
– Teachers with peripheral positions in the teaching profession (i.e., replacement teachers and those teaching in lower-secondary schools or primary schools) have lower self-assessed office digital skills than those with positions seen to be more important. However, the same is not true in the case of multimedia and online skills. There is also a suppression involving age and position in the profession: positions that indicate a higher status in the education field (i.e., tenure, grade, or being taught in an urban area) suppose better digital office skills; however, simultaneously, they are negatively related to age, therefore controlling for status in the education field makes the impact of age on digital office skills more evident.

Considering all these conclusions to the present study, it is clear that post-pandemic Romania’s pedagogical paradigms must be rethought. Teachers must be aware that digital competences will be integrated into professional competences, and they must proactively act and reflect on their development of this critical skill set. For education managers and local authorities, it is a priority to ensure a high-quality education through providing the material resources (i.e., computers, internet connection, and devices for all children and teachers) and sustaining the in-service courses for teachers and staff about the possibilities of adapting to this contemporary challenge (Simuţ et al., 2021). For education policy-makers, it is necessary to analyze all available information and make forward-thinking decisions regarding both national and European strategies about European key competences; teachers’ digital competences; and digital education strategy, monitoring, and implementation (Commission/Eurydice, 2019).

For teachers, using ICT in their professional practice can be considered an efficient and effective means of facilitating access, storage, transmission, and manipulation of different information sources and content *via* audio and video due to its capacity to establish a proactive teaching and learning environment. The education community is currently fundamentally affected by the impact of a new, pressing need for communication and information technologies that are more and more integrated into pedagogical practices and methods, allowing for the consideration of new directions, improvements, or even transformations (Ali, 2020). Indeed, ICT in education can be used for a wide range of different purposes, such as active teaching and learning through Students’ involvement (Ghavifekr and Quan, 2020), or helping in lesson planning or the daily life of teachers (Yevelson-Shorsher and Bronstein, 2018). Ultimately, it is necessary to develop technological resources, especially digital tools for education, and to prepare both students and teachers to be digitally adapted by utilizing ICT with efficiency and respect for ethical principles.

## Data Availability Statement

The original contributions presented in the study are included in the article/supplementary material, further inquiries can be directed to the corresponding author/s.

## Ethics Statement

The studies involving human participants were reviewed and approved by the Stefan cel Mare University. The patients/participants provided their written informed consent to participate in this study.

## Author Contributions

All authors listed have made a substantial, direct, and intellectual contribution to the work, and approved it for publication.

## Conflict of Interest

The authors declare that the research was conducted in the absence of any commercial or financial relationships that could be construed as a potential conflict of interest.

## Publisher’s Note

All claims expressed in this article are solely those of the authors and do not necessarily represent those of their affiliated organizations, or those of the publisher, the editors and the reviewers. Any product that may be evaluated in this article, or claim that may be made by its manufacturer, is not guaranteed or endorsed by the publisher.
